# Effects of Hand Configuration on the Grasping, Holding, and Placement of an Instrumented Object in Patients With Hemiparesis

**DOI:** 10.3389/fneur.2019.00240

**Published:** 2019-03-19

**Authors:** Ross Parry, Sandra Macias Soria, Pascale Pradat-Diehl, Véronique Marchand-Pauvert, Nathanaël Jarrassé, Agnès Roby-Brami

**Affiliations:** ^1^Institut des Systèmes Intelligents et de Robotique, Sorbonne Université, Paris, France; ^2^Centre de Recherche sur le Sport et le Mouvement, EA 2931, Université Paris Nanterre, Nanterre, France; ^3^Service de Médecine Physique et de Réadaptation, Assistance Publique-Hôpitaux de Paris, Hôpitaux Universitaires Pitié Salpêtrière-Charles Foix, Paris, France; ^4^AP-HP, GRC n°18 Handicap cognitif et réadaptation (HanCRe), Sorbonne Université, Hôpitaux Universitaires Pitié Salpêtrière-Charles Foix, Paris, France; ^5^Laboratoire d'Imagerie Biomédicale, Sorbonne Université, Paris, France

**Keywords:** hand function, grasp, stroke, assessment, instrumented objects for rehabilitation

## Abstract

**Objective:** Limitations with manual dexterity are an important problem for patients suffering from hemiparesis post stroke. Sensorimotor deficits, compensatory strategies and the use of alternative grasping configurations may influence the efficiency of prehensile motor behavior. The aim of the present study is to examine how different grasp configurations affect patient ability to regulate both grip forces and object orientation when lifting, holding and placing an object.

**Methods:** Twelve stroke patients with mild to moderate hemiparesis were recruited. Each was required to lift, hold and replace an instrumented object. Four different grasp configurations were tested on both the hemiparetic and less affected arms. Load cells from each of the 6 faces of the instrumented object and an integrated inertial measurement unit were used to extract data regarding the timing of unloading/loading phases, regulation of grip forces, and object orientation throughout the task.

**Results:** Grip forces were greatest when using a palmar-digital grasp and lowest when using a top grasp. The time delay between peak acceleration and maximum grip force was also greatest for palmar-digital grasp and lowest for the top grasp. Use of the hemiparetic arm was associated with increased duration of the unloading phase and greater difficulty with maintaining the vertical orientation of the object at the transitions to object lifting and object placement. The occurrence of touch and push errors at the onset of grasp varied according to both grasp configuration and use of the hemiparetic arm.

**Conclusion:** Stroke patients exhibit impairments in the scale and temporal precision of grip force adjustments and reduced ability to maintain object orientation with various grasp configurations using the hemiparetic arm. Nonetheless, the timing and magnitude of grip force adjustments may be facilitated using a top grasp configuration. Conversely, whole hand prehension strategies compound difficulties with grip force scaling and inhibit the synchrony of grasp onset and object release.

## Introduction

Cerebrovascular accidents (stroke) are a frequent cause of disability (1) and the recovery of upper-limb function in particular, is a key determinant of independence in activities of daily living (2). Broadly speaking, the physical impairment experienced by patients is characterized by loss of strength, abnormal movement patterns (pathological synergies), and changes in muscle tone to the side of the body contralateral to the stroke (3, 4). This presentation is commonly referred to as hemiparesis and its severity tends to reflect the extent of the lesion to the corticospinal tract (5). Subtle changes in movement kinematics and hand function on the ipsilesional upper-limb have also been documented and may be the consequence of direct impairment of ipsilateral motor pathways (6, 7), as well as reorganization of the non-lesioned hemisphere to support recovery of motor-function in the hemiparetic limb (8–10). Above all though, patients living with stroke find that limitations with manual dexterity of the hemiparetic arm have the most significant effect upon their ability to carry out activities involving hand use in daily life (11).

These impairments in patient hand function manifest in multiple different aspects of motor performance. This may include reduced strength (3), loss of individuated finger control (12), and abnormal force control at the level of the fingers (13). Increased muscle tone and spasticity though the flexors of the wrist and hand may further compound these difficulties and inhibit the ability to open the hand in preparation for grasping (14). Atypical reaching and grasping patterns are often seen to emerge both as a consequence of and as a response to the motor dysfunction (15, 16).

Unfortunately, rehabilitation of upper limb impairments proves to be challenging. Whilst numerous therapeutic modalities (e.g., bilateral training, constraint-induced therapy, electrical stimulation, task-oriented, high intensity programs) have been evaluated in clinical trials, none have demonstrated consistent effects upon hand function (17–19). Indeed, previous research papers have described therapy outcomes in upper limb rehabilitation post stroke as “unacceptably poor” (20). Ideally, the design of neurorehabilitation programs should be grounded upon an understanding of basic mechanisms involved in neural plasticity and motor learning (21, 22). Part of this process implies coming to terms with the factors which characterize the disorganization in voluntary motor output (21). However, the majority of clinical tools currently used for evaluating hand function distinguish motor performance according to ordinal rating scales or task completion time (e.g., Frenchay Arm Test, Jebson-Taylor Hand Function Test) (23, 24). These kinds of assessments lack sensitivity and may prove insufficient for detecting the presence of mild motor deficits or subtle, yet clinically important changes in hand coordination (25, 26). Evidence based frameworks for hand rehabilitation have specifically called for the integration of new technology to support patient assessment and treatment planning (27). Despite this, the transposition of technology for upper limb rehabilitation from the research domain into clinical practice has been limited (28, 29). In the assessment of manual dexterity, the underlying challenge involves analyzing sensorimotor function of the hand with respect to its interaction with objects in the environment (30).

Successfully managing grasping and object handling tasks requires skilled control of prehensile finger forces. In healthy adults, grip forces are regulated to be marginally greater than the minimum required to prevent the object from slipping (31). This safety margin is calibrated according to the shape, surface friction and weight distribution of the object (32, 33). As the hand moves through space (lifting, transporting, object placement), grip force is continually modulated, proportional to the load forces associated with the mass and acceleration of that object (34). This temporal coupling between grip and load forces is considered a hallmark of anticipatory sensorimotor control (35). Disruption to motor planning, volitional motor control or somatosensory feedback may lead to a breakdown in the timing and magnitude of grip force adjustments.

Numerous studies have examined grip force regulation in neurological pathologies including cerebellar dysfunction (36), peripheral sensory neuropathy (37, 38), Parkinson's disease (36, 37, 39, 40) as well as congenital and acquired brain lesions (13, 36, 41–45). For patients suffering from hemiparesis post stroke, difficulty with coordinating the grasping and lifting action are frequently associated with temporal discrepancies between grip forces and load forces (46). The cerebral hemisphere implicated in the CVA (13, 47) and the extent of the resulting sensory deficits (48, 49) have also been observed to influence anticipatory grip force scaling. This body of work highlights the potential interest of using instrumented objects for the diagnosis and evaluation of the impairments associated with hemiparesis (45, 46, 48, 50–53).

As it stands, these objective studies of hand function post stroke have focused primarily upon either the lifting or the vertical movement components in object handling. To a certain extent, this limitation has been related to technical restrictions. Other than a handful of studies by Hermsdorfer et al. (8, 49), research in this field has predominantly used manipulanda designed for the study of precision grip, where strain gauge force transducers are attached to a separate base unit [e.g., (23–25, 29, 33, 35, 37)]. These devices cannot be freely handled by subjects, much less a person with an upper-limb movement disorder. Indeed, patients with hemiparesis often experience specific impairments with precision grip (53) and regularly use alternative grasping strategies such as whole hand grasping (15, 16, 54). Previous researchers have hypothesized that these alternative grasp strategies may impact grip force scaling (55) and compromise patient ability to manage hand-object-environment relationships during object manipulation (56).

In a recent study with healthy adult subjects, (57) we demonstrated how an instrumented object with multiple load cells and an integrated inertial measurement unit (58) may be used to examine relationships between different grasp configurations, grip force regulation and object orientation. The purpose of the present investigation was to extend this work to the study of patients with hemiparesis post stroke. The first objective was to compare how four alternative grasp configurations commonly used in daily tasks affect grip force regulation in this population. The second objective was to explore the timing and coordination of the whole task sequence (grasping, lifting, holding, placement and object release). The third and final objective was to evaluate the stability of the hand-held object's orientation across the different phases of the task.

## MaterialS and Methods

### Participants

Twelve adult patients (6 males, 6 females) with a diagnosis of a unique stroke and a mean age of 58 years (range 48–70 years) participated in this study. Of these patients, 8 suffered from hemiparesis on their dominant right hand side; 4 right handed patients and 1 ambidextrous patient suffered from left sided hemiparesis [hand preference verified using the Edinburgh Handedness Inventory, see (59)]. Each patient was in a subacute or chronic phase of recovery and was assessed between 1 and 13 months following the neurological event. The ability to grasp and hold an object was a requirement for inclusion to this study. Patients with additional neurological or orthopedic conditions, important cognitive deficits or aphasia were not eligible for this study. A summary of clinical characteristics of the patient group is provided in Table 1. This study was approved by the local ethics committee at University Paris Descartes and all subjects provided written consent prior to commencement of the evaluation.

**Table 1:** Results from the functional upper limb evaluations for stroke patients.

**Patient ID**	**Hemiparetic arm**	**Time since stroke**	**Dynamometer grip strength (reference from less-affected side)**	**Fugl-Meyer upper limb evaluation (sensory/motor subscores)**	**Jebsen Taylor hand function test (reference from less-affected side)**	**Frenchay arm test**
P1	Right (d)	5 months	361.6 N (353.1 N)	124 (58/66)	79 s (80 s)	5
P2	Right (d)	13 months	156.8 N (473.3 N)	95 (39/56)	303 s (95 s)	3
P3	Right (d)	11 months	215.6 N (363.6 N)	105 (56/49)	89 s (84 s)	5
P4	Right (d)	2 months	38.2 N (197.0 N)	84 (42/42)	337 s (110 s)	5
P5	Right (d)	18 months	245.9 N (382.8 N)	105 (56/49)	261 s (163 s)	5
P6	Left (n)	1 months	107.8 N (367.2 N)	109 (53/56)	308 s (52 s)	4
P7	Left (n)	2 months	52.9 N (235.9 N)	78 (41/37)	362 s (45 s)	3
P8	Right (d)	19 months	146.0 N (189.4 N)	124 (59/65)	61 s (65 s)	5
P9	Left (n)	5 months	26.5 N (156.8 N)	104 (38/66)	NA	3
P10	Left (a)	13 months	266.6 N (275.4 N)	120 (60/60)	NA	5
P11	Right (d)	2 months	332.2 N (381.2 N)	125 (65/60)	NA	5
P12	Right (d)	14 months	16.7 N (124.5 N)	96 (48/48)	NA	5
***n* = 12**	**8 right/4 left**	**9 months**	**163.7 N (291.7 N)**	**106 (51/55)**	**225 s (87 s)**	**5**

*For the hemiparetic arm, (d), (n), (a) signify if this is the dominant, non-dominant or ambidextrous hand. Grip strength scores are provided in newton with values for the less affected side in brackets. Fugl-Meyer provides the total score on the upper limb evaluation with sensory and motor subscores indicated in brackets. The Jebsen Taylor provides a score in seconds, being the total time required to complete a series of manual handling tasks; the score in brackets provides the reference time for the less affected arm*.

### Clinical Measures of Upper-Limb Function

Prior to completing the experimental phase of this study, an upper-limb motor-function assessment was carried out. The Fugl-Meyer upper-limb evaluation (FME) and Frenchay Arm Test (FAT) was conducted for each patient and, in addition to this, 8 of the 12 patients completed the Jebsen Taylor Hand Function test (JTT). The FME evaluation provides an overall score of upper limb function (max of 126), which may then be broken down into its sensory function component (max of 60) motor function component (max of 66) (60). The FAT assesses patient ability to carry out five different actions providing a score on a scale of 1 to 5 (61). The JTT provides an overall score in seconds, representing the time taken to complete a series of functional task with each arm. Finally, hand strength for both arms was measured using a grip-strength dynamometer (DGS).

### Experimental Apparatus

**Figure 1:**
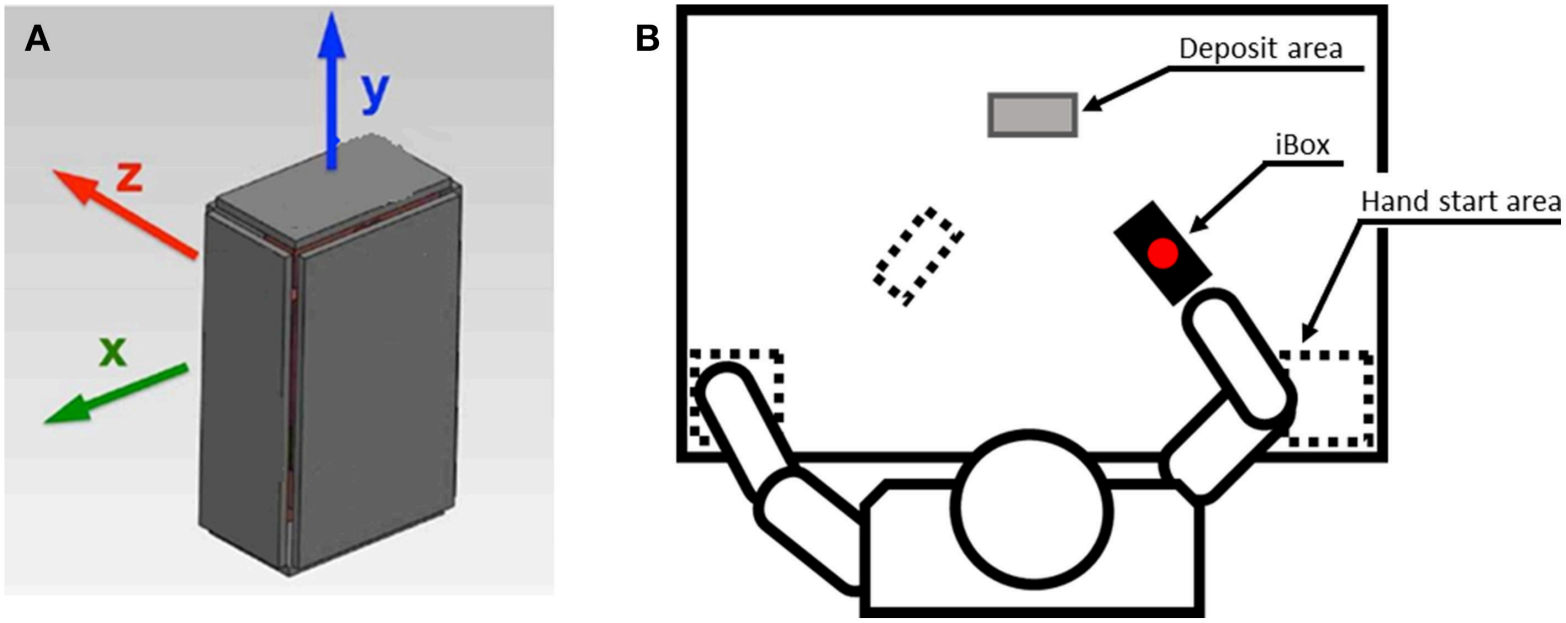
Illustration of the iBox device and the experimental setup. **(A)** The iBox instrumented object. **(B)** Setup for the experimental procedure. Initial positions of the iBox and hand start area are indicated by the dotted lines. The gray shaded rectangle indicates the deposit area for the top grasp task.

An instrumented object (iBox) with 6 integrated load cells and an inertial measurement unit (IMU) was used for the purposes of this study (see Figure 1A). This device measures 108 × 70 × 40mm and has a mass of 0.370 kg. It enables recording of acceleration, rotational velocity, orientation of the unit as well as the forces applied normally to each of its six faces. The force of the load cell on the bottom face was calibrated so that the weight of the device, equivalent to 3.63 N, was subtracted (i.e., the reference force signal was zero when the object lay on the table and decreased to −3.63 N when the object was lifted from the supporting surface). All data was sampled at a frequency of 100 Hz and transmitted wirelessly to a local computer via Bluetooth. Overall acceleration was measured as a combination of gravity and kinematic acceleration (39). Object orientation was calculated from IMU data and expressed as the alpha angle, indicating the deviation of the longitudinal axis of the iBox from the vertical axis. Further technical details regarding the iBox are provided in (58).

### Installation

Subjects were seated at a horizontal table throughout the experiment. In the starting posture, both hands were positioned at each corner of the proximal edge of the table. The iBox was placed vertically before the patient. It was positioned in the parasagittal plane, 20 cm in front of the hand used for the pinch, precision and top grasps. For the palmar digital grasp, the iBox was placed in front of the opposite hand so as to ensure a comfortable grasp (15, 57). In all cases the iBox was rotated 30° around the vertical axis, in the direction of the patient's midline. This reference orientation was calibrated at the beginning of the experiment and repeated prior to each trial. The experimental setup is illustrated in Figure 1B.

### Grasp Configurations Used

**Figure 2:**
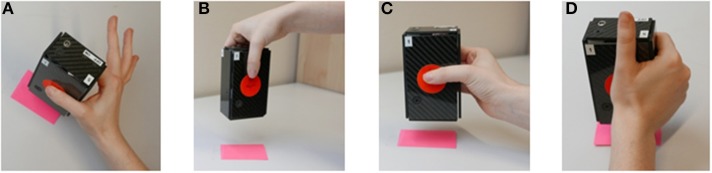
Grasp configurations used during the iBox protocol. **(A)** Precision grip. **(B)** Top grasp. **(C)** Pinch grip. **(D)** Palmar-digital grasp. Image adapted from Martin-Brevet et al. (57).

The experimental procedure involved grasping and holding the iBox using 4 different hand configurations. Each of these grasps, described below is illustrated in Figure 2.

- *Precision grip:* opposition between the pads of the thumb and index (Figure 2A).
- *Top Grasp:* opposition using a pinch grip, the object is approached and grasped from above (Figure 2B).
- *Pinch grasp:* opposition between the pads of the thumb and palmar aspect of the four fingers (Figure 2C).
- *Palmar-digital grasp:* opposition of fingers and palm, with the thumb in abduction as for a power grip (Figure 2D).

This combination of grasps was selected to represent common hand configurations which may support functionally different tasks in daily activities. For example, pinch grasps are a versatile hand configuration that can support an object whilst enabling transition to in-hand manipulation if necessary, while precision grasps are important for handling smaller objects. By contrast, a palmar digital grasp serves to fix an object in the hand while the arm is in motion (i.e., scrubbing a surface with a sponge) whereas the top grasp configuration may assist with tasks such as repositioning objects on a table's surface [see (62) for greater detail on the frequency of grasp configuration in household tasks].

### Experimental Procedure

Each patient was given a brief period of time to handle the iBox with both hands prior to beginning the experimental tasks in order to become familiar with the weight and surface characteristics of the object. During the experimental task, patients were asked to lift and hold the iBox approximately 10 cm above the table. For the pinch, precision and palmar-digital hand configurations, patients were instructed to hold the iBox for between 2 and 5 s before replacing it in an approximately similar position. For the top grasp configuration, patients were asked to place the iBox in the frontal plane, 10 cm distal to the initial position (deposit area indicated in Figure 1) (57). A demonstration was provided prior to commencement of each task. Patients were asked to perform each grasp and place task 3 times to the best of their ability. The ensemble of grasping and holding tasks were performed first with the less affected arm and then with the hemiparetic arm. The experimenter verified the patient's initial posture and repositioned the iBox between movements as required.

Visual inspection of all force, acceleration and orientation signals was carried out immediately following data acquisition. Events where signals were compromised or patients were unable to complete the set task were excluded. All patients were able to perform the palmar and top grasp tasks with both limbs. Using the hemiparetic arm, one patient (patient 9) was unable to perform the pinch grasp task and four patients (patients 3,6,7,9) were unable to complete the precision grip task.

### Data Processing and Analysis

**Figure 3:**
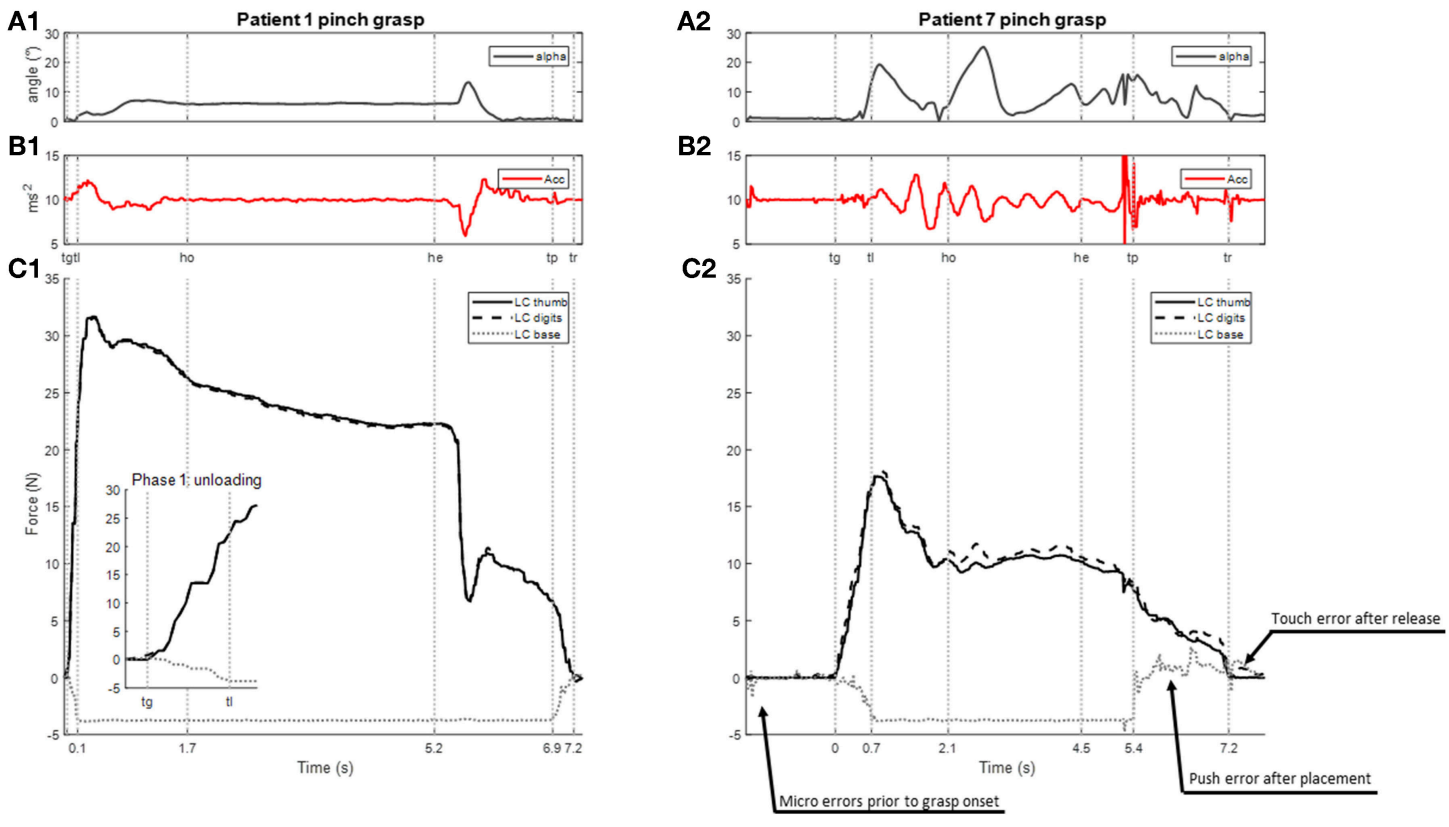
Examples of recording of a lifting task carried out with the hemiparetic arm using the pinch grip in two patients with contrasting functional abilities (P1, FME 66 and P7 FME 37; see Table 1 for details). From top to bottom: **(A1,2)** angle measuring the deviation of the iBox from the vertical **(B1,2)** vertical acceleration of the iBox **(C1,2)** force signals: grasping force is indicated with plain (thumb) and dashed lines (digits), the unloading of the bottom face of the object is indicated with (dotted lines); inset in **(C1)** shows a larger scale. Vertical lines indicate the times of transitions between phases *tg* = onset of grip; *tl* = onset of lifting; *ho* = hold onset; *he* = hold end; *tp* = placement time; *tr* = release time. Time = 0 s at *tg*. In **(C2)**, arrows indicate touch and push errors upon establishing and releasing grasp.

Transitions between grasping, lifting, and placement phases were identified in an automated manner with reference to load cell data (57) (Figure 3 indicates the different phase transitions with vertical lines). *Grasp onset* (*tg*) was defined as the moment when the mean of the forces applied to the two lateral load cells exceeded 0.15 N. O*nset of lifting* (*tl*), when the base load cell value was inferior to the −3.4 N threshold. *Placement time* (*tp*) was the moment when the base load cell then returned to the threshold value of −3.4 N. *Object release time* (*tr*) was defined as the moment when the mean of the forces applied to the two lateral load cells were inferior to 0.15 N. The *hold onset* (*ho*) and *hold end* (*he*) events were chosen subjectively to delimit a plateau of relative stability during holding and tagged manually from data in each trial using a graphic interface. From these events, five separate phases were identified: (1) *unloading* of the bottom face between *tg* and *tl*^1^, (2) *lifting* between *tl* and *ho*, (3) *holding* between *ho* and *he*, (4) *descent* between *he* and *tp*, and (*5*) *release* between *tp* and *te*.

Further to this, the occurrence of *push* and *touch* errors (57) were identified. Touches were identified where extraneous forces were applied to the object prior to grasp onset or following object release. A touch was defined as an event where the sum of forces on the exposed (front, back, top, and lateral) load cells exceeded 0.7 N before *tg* or after *tr* for any given trial. The first face of the object touched was identified and noted. A push was detected as increased force (>0.4 N) on the base load cell during the unloading or release phases. Examples of touch and push events are illustrated in the load cell signals provided in Figure 3C2.

Based upon the time-tagged data sequences, the following series of variables were extracted for analysis:

- Duration and rate of grip force change for unloading and release phases
- Grip force at *tg, tl, tp, te* (mean of the front and back load cells)
- Maximal grip force and peak acceleration during the lifting phase
- Time difference between maximal grip force and peak acceleration during the lifting phase
- Grip force during holding (median and standard deviation of the *front and back load cells* during the whole period)
- iBox orientation at times *tg, tl, td, te* (alpha angle)
- iBox orientation during holding (alpha angle median and standard deviation)
- Frequency of touch events before grasping and after object release and of push events during the unloading and release phases

All data analysis was performed using customized Matlab scripts.

### Statistical Analysis

Data for continuous variables were examined using Shapiro-Wilk tests. As the ensemble of these variables was found to have non-normal distributions, Kruskall-Wallis non-parametric analysis of variance was used for statistical comparisons. Both side (hemiparetic arm/less-affected arm) and grasp configuration (pinch/precision/palmar digital/top) factors were included. Where indicated, *post-hoc* analysis was conducted using Dunn's method. The frequency of touch and push errors was analyzed using Chi-Squared tests. The Bonferroni method was used for correction of *p*-values when comparing across grasp configurations. The threshold for statistical significance was set at *p* = 0.05.

In order to evaluate relationships between clinical characteristics and task performance, test results from the DGS, FME, JTT, and FAT were transformed into z-scores prior to testing with Spearman correlation coefficients against the hemiparetic upper-limb variables assessed using the iBox. Values >0.7 or <-0.7 were considered to represent strong correlation between clinical motor-function tests and iBox variables. In order to control for multiple correlation analysis, a resampling method with 10,000 randomized permutations of each iBox variable was used. Percentile values (2.5 and 97.5%) from the distribution of the resulting coefficient matrix served as a symmetric two-sided 95% confidence interval (63). Correlations of clinical motor tests and iBox variables outside of this confidence interval were considered as statistically significant. All statistical analyses were conducted using Matlab and the JASP software package (https://jasp-stats.org).

## Results

### Clinical Measures of Upper-Limb Function

Average grip-strength for the affected arm was 163.7 N (s.d. 120.5 N; range 16.7–361.6 N) compared to 292.0 N (s.d. 109.8 N; range 124.46–473.3 N) for the less affected arm. The patient group was assessed as having mild to moderate upper-limb impairment using the FME motor assessment (median = 56; range 37–66) with variable levels of sensory deficits (range 38–60 on the sensory function subscore). The median score on the Frenchay Arm Test was 5 (range 3–5), indicating that patients were able to carry out basic functional tasks with their affected upper-limb. The median time for completion of the JTT with the hemiparetic arm was 282 s (range 61–362 s), vastly superior to that of average times for similarly aged individuals (average 30 s, (64, 65). Clinical measures of upper-limb function are displayed in Table 1.

### Time Courses for iBox Data Signals

Time courses of force, acceleration and object orientation signals were generally consistent across the different grasp patterns used. Changes in grip forces reflected the phase progression in the grasping, lifting, holding and placement of the iBox, although the regularity and magnitude of these signals were less consistent. Figure 3 provides typical examples of these signals for two patients with contrasting functional abilities (patient 1 had a FME motor score of 66 compared to 37 for patient 7). Broadly speaking, those patients who experienced a better recovery had regular acceleration and orientation profiles. For these patients, maximal grip force occurred during lifting and a smooth decrease of force was observed before placement while the holding phase was characterized by relative stability of grip forces. Patients with more severe motor deficits demonstrated greater variability in the acceleration and object orientation profiles (see examples in Figures 3A1,2,B1,2). In the following section, the main results of this experiment are presented according to the five phases (unloading, lifting, holding, descent, release) which characterize the task.

### Unloading Phase

Grip force at *tg* was found to vary with grasp configuration (Kruskal Wallis, *p* = 0.011) and *post-hoc* testing showed that force in the palmar-digital grasp was greater than in the precision (*p* = 0.009) and top grasps (*p* = 0.018).

**Figure 4:**
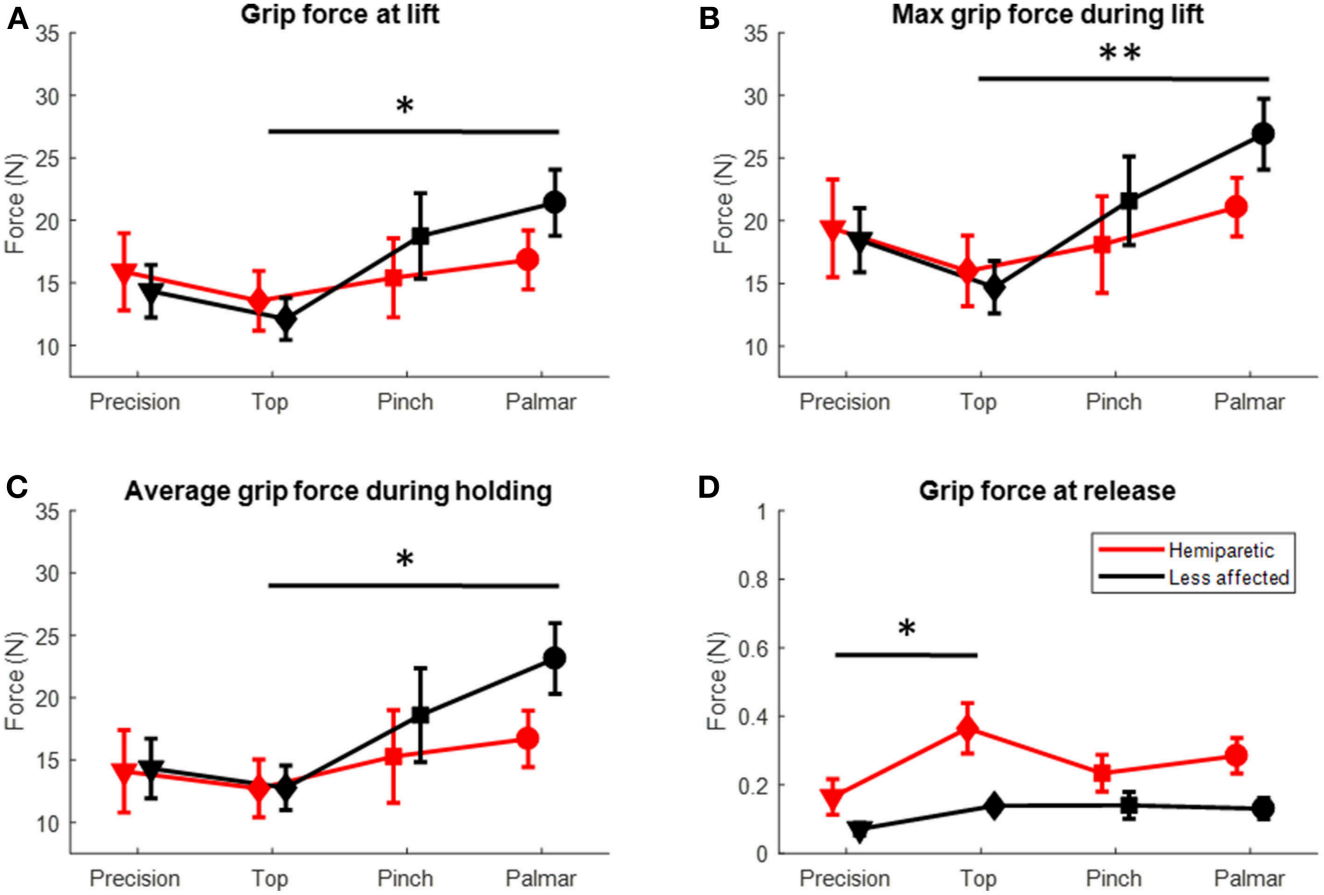
Grip forces for the hemiparetic (red symbols) and less affected arms (black symbols) for the different grasp configurations (in abscissa). **(A)** Grip force at the time of lifting (tl). **(B)** Maximum grip force during the lifting phase **(C)** Average force during the holding phase **(D)** Grip force at the time of release.*Dunn's *post-hoc p* < 0.05; **Dunn's *post-hoc p* < 0.01.

**Figure 5:**
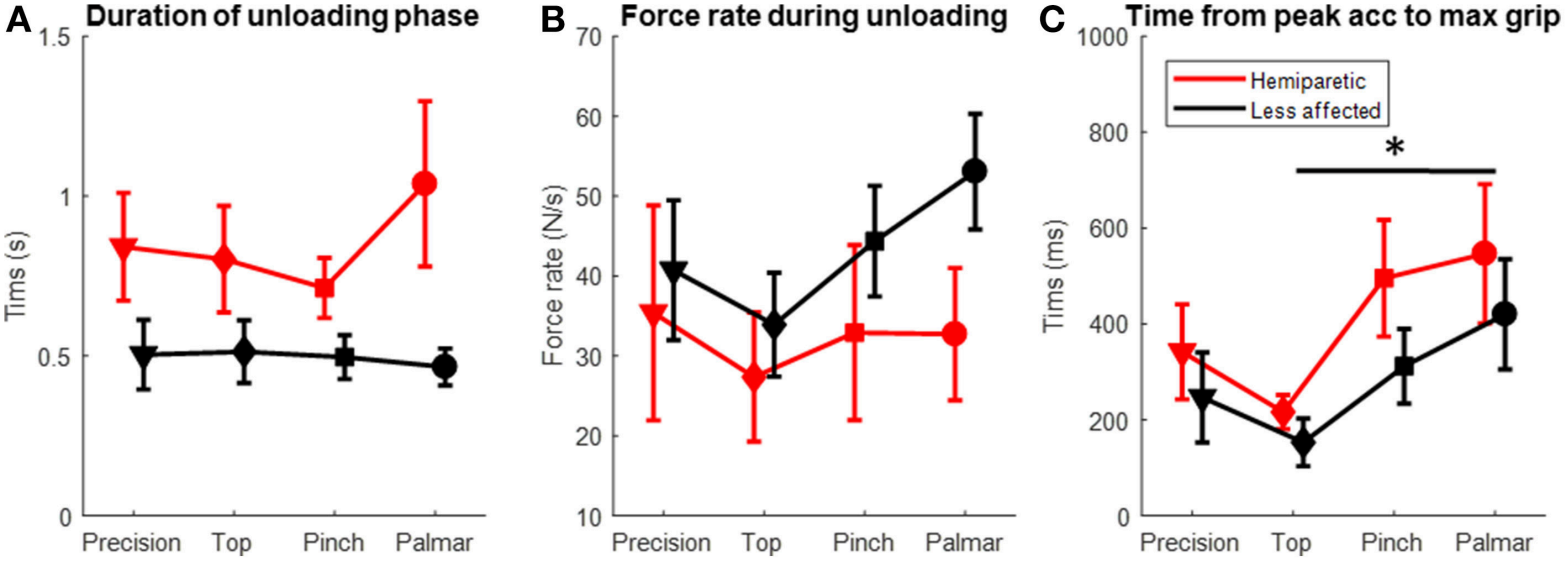
Temporal data for unloading and lifting phases in the hemiparetic (red symbols) and less affected arms (black symbols) using different grasp configurations (in abscissa). **(A)** Duration of the unloading phase. **(B)** Time difference between maximal grip force and peak acceleration during the lifting phase. **(C)** Rate of grip force change during the unloading phase. *Dunn's *post-hoc p* < 0.05.

The subsequent unloading phase was characterized by a progressive increase in grasp forces and a corresponding decreased load on the base of the instrumented object until *tl* when it reached −3.63 N (see examples in Figures 3C1,2). At *tl*, grip force was found to vary with grasp configuration (Kruskall-Wallis *p* = 0.038, Figure 4A). Grip forces were significantly lower when using the top grasp (average of 12.85 N) than when using a palmar-digital grasp (average of 19.03 N; *p* = 0.013). The overall duration of the unloading phase was greater when using the hemiparetic arm (0.85 s on average) than the less-affected arm (0.49 s on average) (Kruskal Wallis, *p* = 0.002; Figure 5A) and grip force rate was accordingly diminished on the hemiparetic side (Kruskal Wallis, *p* = 0.003; Figure 5B).

**Figure 6:**
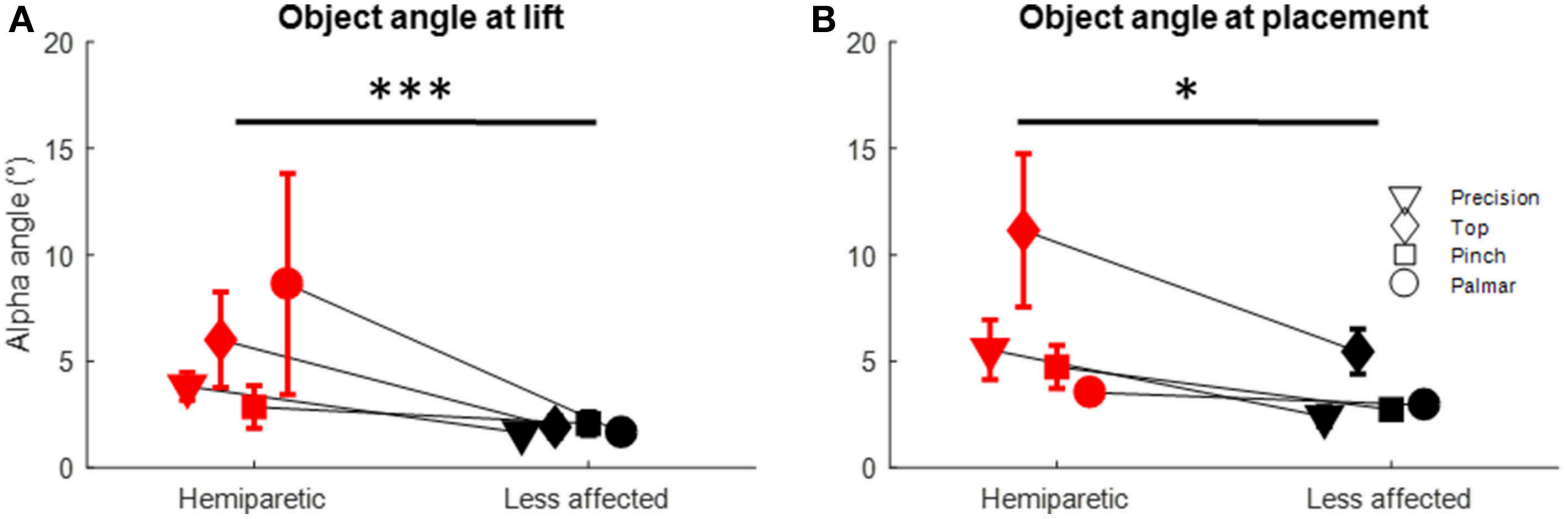
Object orientation for the hemiparetic (red symbols) and less affected arms (black symbols) at: **(A)** Time of lift and, **(B)** Time of placement. *Dunn's *post-hoc p* < 0.05; ***Dunn's *post-hoc p* < 0.001.

The mean orientation of the iBox at *tl* was 5.4° on the hemiparetic arm, significantly greater than that of the 1.8° for the less affected arm (Kruskall-Wallis *p* = 0.001; Figure 6A).

**Figure 7:**
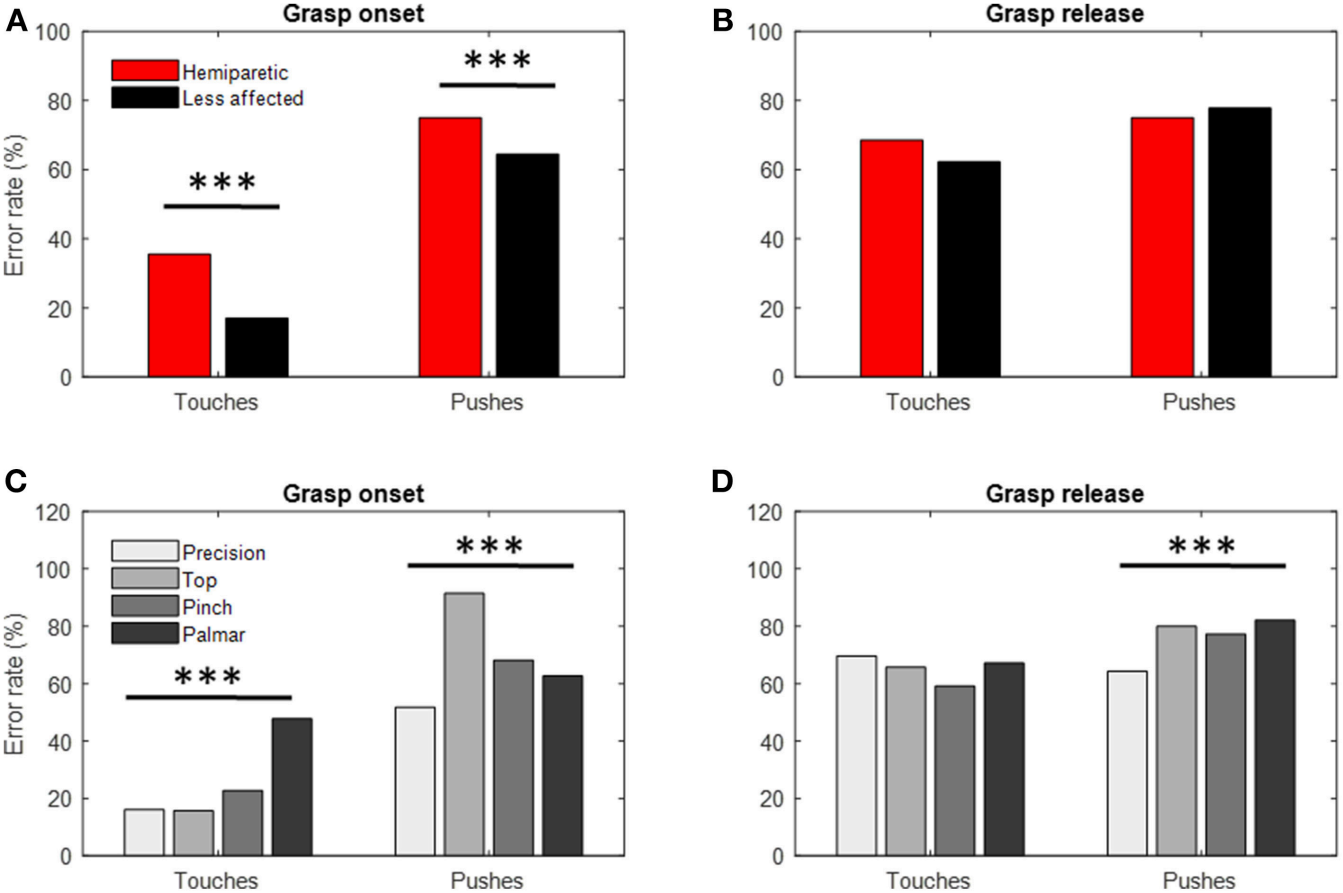
Frequency of touch and push errors. **(A)** Frequency of touch and push errors made at grasp onset by the hemiparetic (red) and less affected (black) arms. **(B)** Same data distributed according to the different types of grasps used. **(C)** Frequency of touch and push errors made at grasp release by the hemiparetic (red) and less affected (black) arms. **(D)** Same data distributed according to the different types of grasps used. ***Chi-squared test *p* < 0.001.

The occurrence of touch and push errors varied with both the grasp configuration and the arm used (Chi-Squared *p* < 0.001; per Figure 7). Touch errors were most frequent when using the palmar (48% of trials) and pinch grasps (23% of trials). This type of error was also twice as frequent in the hemiparetic arm (35% of trials) than in the less-affected arm (17% of trials). When using the hemiparetic arm, these errors were associated predominantly with sub-threshold touches on the load cell corresponding to finger contact (18%) than for the load cell corresponding to the thumb (8%). On the unaffected arm, this trend was reversed with many more errors attributed to sub-threshold contact from the thumb (10%) than for the fingers (2%). Push errors occurred more systematically than touch errors. They occurred most frequently with the top grasp (91% of trials) and pinch grasps (68% of trials). Again, these errors were more common for the hemiparetic arm (75% of trials) than for the less-affected arm (64% of trials).

### Lifting Phase

During the lifting phase, grip forces were generally observed to continue to increase in accordance with the vertical acceleration of the iBox (examples in Figures 3B1–C1, B2–C2). Several patients (1, 10–12) were found to have particularly high maximal grip forces in the lifting phase, to the point where the load cells were saturated (limit of 40 N) on several trials. While no differences were observed for peak acceleration, the maximal grip force through the lifting phase varied with the grasp used (Kruskall-Wallis *p* = 0.009, Figure 4B) and *post-hoc* testing showed that the maximal grip forces were significantly greater for the palmar-digital than for the top grasp (*p* = 0.003).

**Figure 8:**
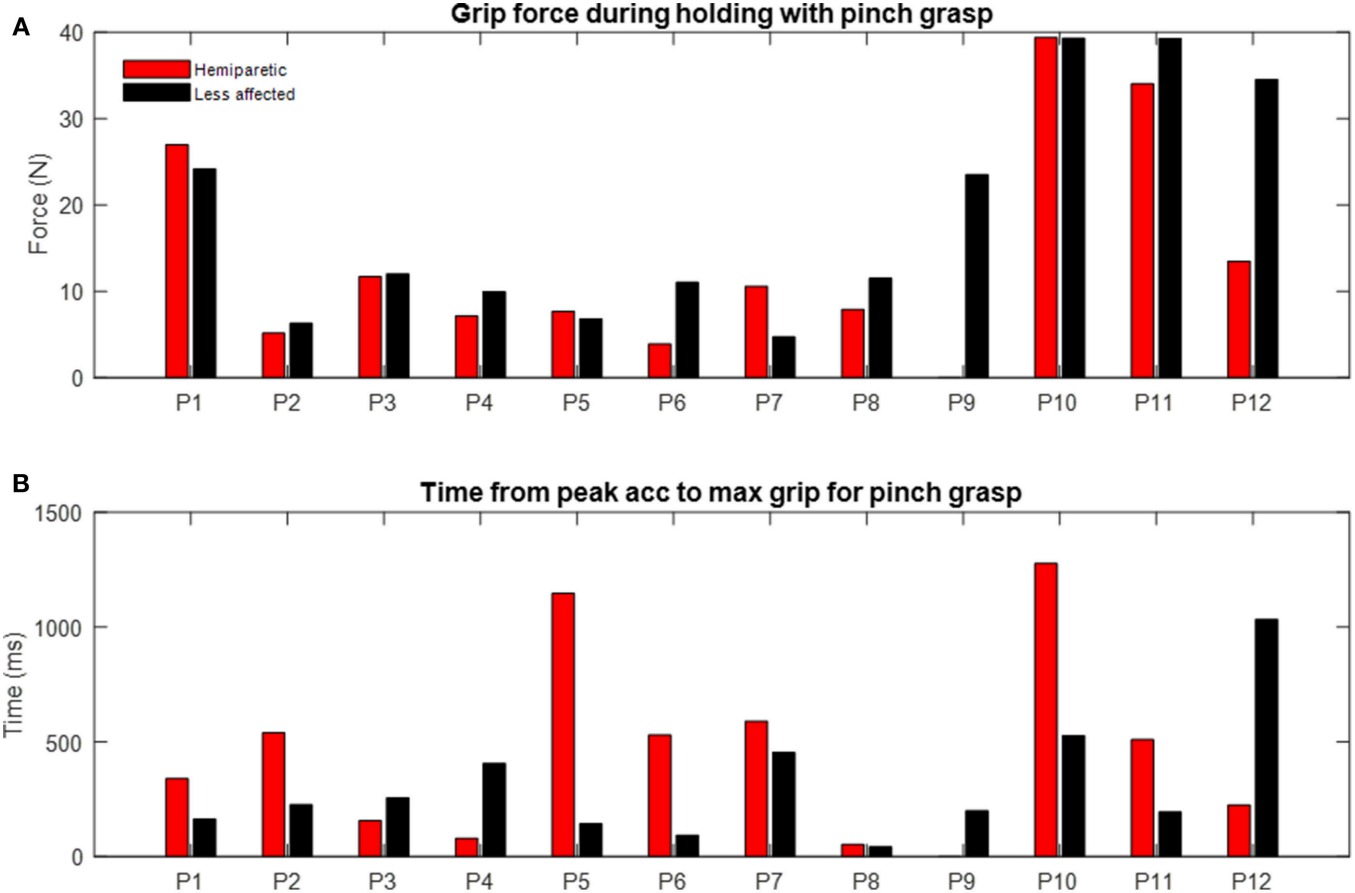
Examples of individual differences during pinch grasp with hemiparetic (red) and less affected arms (black). **(A)** Grip force during holding. Each bar represents median grip force recorded for each patient. **(B)** Time delay from peak acceleration to maximum grip force. Each bar represents mean of time delay over three trials.

Time difference between maximal grip force and peak acceleration varied with grasp configuration (Kruskall-Wallis *p* = 0.02) and the arm used (Kruskall-Wallis *p* = 0.03; see Figures 5C, 8B). For example, the average lag time was 185 ms when using a top grasp, significantly lower than that of 486 ms when using the palmar-digital grasp (*p* = 0.02).

### Holding Phase

Grip forces during the holding phase were observed to be particularly variable from one individual to another (s.d. 9.70 N; range 3.92–40 N). In the examples provided in Figure 3, the grip force during holding for patient 1 (panel C1) is more than twice as great as the grip force for patient 7 (panel C2) for the same grasp and place task using the pinch grip. Three patients (10–12) were again observed to saturate load cells during this phase. Figure 8A provides a comparison of average grip force during holding when using the pinch grasp. Overall, grip force during holding was found to vary in relation to grasp configuration (Kruskall-Wallis *p* = 0.027; see Figure 4C). On average, grip force when holding with the top grasp was 12.75 N, significantly lower than holding with a palmar-digital grasp at 19.77 N (*p* = 0.022).

### Descent and Placement

In the descent phase, average object orientation and standard deviation were observed to vary with grasp configuration (Kruskall-Wallis *p* < 0.001; *p* = 0.007), *post-hoc* testing confirmed that these variables were greater for top grasp than for pinch (*p* = 0.011; *p* = 0.037), precision (*p* = 0.001; *p* = 0.047) and palmar-digital grasps (*p* = 0.003; *p* = 0.004).

Upon placement of the iBox, certain patients appeared to control downward acceleration smoothly, whereas others exhibited important variations in acceleration around the time of placement, *tp*, suggesting vibrations due to the impact of the object on the table (see examples in Figures 3B1,2). Despite this, no significant differences in grip force at *tp* were found.

The deviation of the object from the vertical was greater when using the hemiparetic arm (alpha angle at *tp* of 6.38°) than for the less affected side (alpha angle at *tp* of 3.45°) (Kruskall Wallis *p* = 0.012; see Figure 6B). Grasp configuration was also found to influence object orientation at *tp* (Kruskal-Wallis *p* = 0.003). When using top grasp, alpha angle was 8.18° on average, significantly greater than for the precision (*p* = 0.008), pinch (*p* = 0.06) and palmar-digital grasps (*p* = 0.007).

### Release

During the release phase, the force on the bottom face of the object increased while the grip forces decreased. Those patients with better functional ability appeared to perform this transition relatively smoothly (progressive increase of force on bottom face of iBox and progressive decrease in grip forces, see Figure 3C1). The release phase was comparatively more irregular in patients with poorer functional ability and occasionally associated with an impact of the object on the surface of the table in addition to extraneous touch and push errors (see Figure 3C2).

Grip force at *tr* was greater on the hemiparetic side (average of 0.27 N) than on the less-affected side (0.12 N) (Kruskal-Wallis *p* = 0.01; Figure 4D). At the same time, grip force at *tr* was also observed to vary according to grasp configuration (Kruskal-Wallis *p* = 0.032) and *post-hoc* testing showed that these forces were significantly higher in top grasp than in precision grasp (*p* = 0.017).

The occurrence of push errors was found to vary with grasp configuration (Chi-Squared *p* < 0.001), the palmar-digital grasp being associated with the greatest frequency (82% of trials, see Figure 7D).

### Correlation of Clinical Measures for Upper-Limb Function With iBox Variables

**Figure 9:**
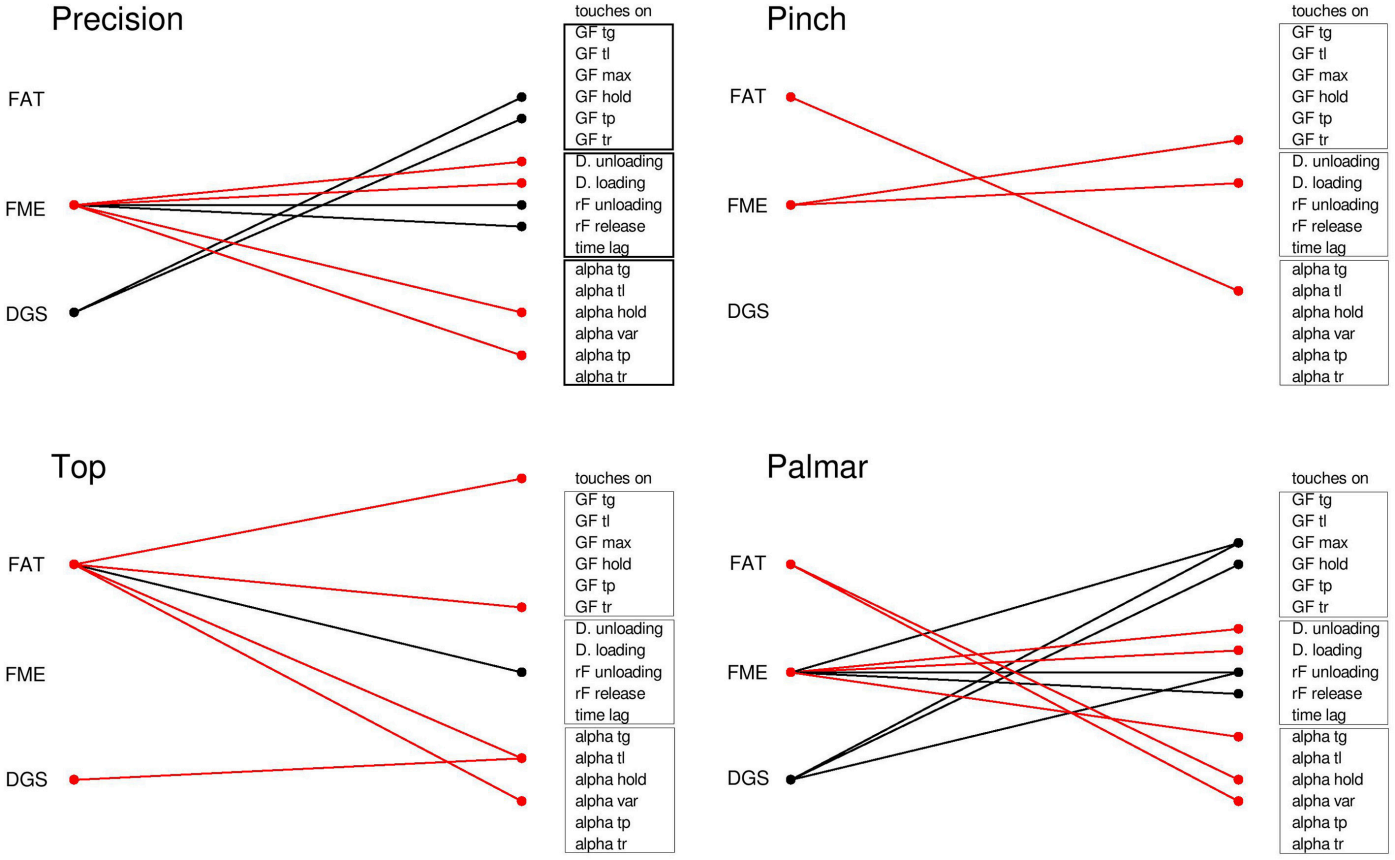
Correlation between clinical data and behavioral variables for the different grasp types. Lines represent significant Spearman correlations (positive in black, negative in red) between clinical measures and iBox variables. FAT, Frenchay arm test; FME, Fugl Meyer Evaluation; DGS, dynamometer grip strength; Touches on, frequency of touches before *tg*; GF, grip force at different time points; D, phase duration; Time lag, time difference between maximal grip force and peak acceleration during lifting; Alpha, deviation of the iBox from the vertical at the different time points; Alpha var, variability of alpha angle during holding.

A summary of statistically significant correlations of dynamometer grip-strength (DGS), Fugl Meyer evaluation (FME) and Frenchay Arm Tests (FAT) scores with iBox variables for each grasp configuration is provided in Figure 9. Each line represents a significant Spearman correlation (black) or negative correlation (red) between a clinical variable (FAT, FMA, and DGS, on the left) and a biomechanical behavioral variable (grouped according force, timing and orientation variables). A table providing all significant correlation data is provided in Tables S1–S6.

For the precision grip, FME was correlated with the temporal parameters of the task (positive correlation with the rate of force change during lifting and placement, inverse correlation with the duration of unloading and placement phases,) and inversely correlated with the angle of the object during holding and at *tp*. Further to this, the FME sensory function subscore was also positively correlated with grip force at several stages of the task (*tl, tp*, maximal grip force, average grip force during holding), while the FME motor subscore was positively correlated with peak acceleration and negatively correlated with the angle of the object during holding (refer to Tables S2, S3, respectively). DGS was correlated with the grip force during holding and at *tp*.

In the case of top grasp, FAT was inversely correlated with touch frequency at grip onset, grip force at *tg*, object angle at *tl* and variability of object angle during holding. It was positively correlated with the rate of force during unloading. The FME motor subscore was negatively correlated with the duration of the unloading and loading phases. The JTT was correlated with temporal parameters during the unloading phase, object angle at *tg* and grip force at *tl* (see Table S5).

For the pinch grip, FAT was inversely correlated with the object angle at *tl* and FME was inversely correlated with the duration of the loading phase and the grip force at *tr*. Both the sensory and motor subscores of the FME were found to be correlated with temporal parameters and force parameters during object release (positive correlation with rate of force change during release, negative correlation with release phase duration and grip force at *tr*). The JTT was correlated with several temporal parameters (positive correlation with release phase duration and lag time from maximal grip force to peak acceleration, negative correlation with rate of force change during unloading and release) as well as being positively correlated with object angle at *tp*.

For the palmar-digital grasp, FAT was inversely correlated with the object angle and object angle variability during holding. FME was correlated with the maximum grip force, the rate of force change during the unloading and loading phases, and inversely correlated with the duration of the unloading and loading phases as well as object angle at *tg*. The FME sensory subscore was negatively correlated with object angle at *tl* and average object angle during the holding phase, while the FME motor subscore was associated with temporal parameters (negative correlation with duration of unloading and loading phases, negative correlation with rate of force change during loading and unloading phases). JTT score was positively correlated with object angle during the holding phase. DGS was correlated with the rate of grip force change during loading, maximum grip force and average grip force during the holding phase.

## Discussion

This study investigated the hand function of stroke patients. Using an instrumented object, several aspects of dexterity were examined: grip force regulation, timing and coordination of the action sequence (grasping, lifting, holding, placement, and release) and stability of the hand-held object. Motor performance was compared across four different grasp configurations commonly used in daily life activities for both the hemiparetic and less affected arms. The results of this study confirmed the hypothesis that grasp configuration has a significant effect upon grip force scaling for patients suffering from hemiparesis (55). The ability to manage object orientation was reduced in the hemiparetic arm when compared to the less affected arm while grasp configuration had comparably less effect.

### Grip Force Regulation During Lifting and Holding

The results of this study are generally consistent with previous research in demonstrating that patients with hemiparesis were globally capable of regulating grip forces with respect to load force variations (8, 22–25, 27, 47). Specific impairments manifested as irregularities in the magnitude and timing of grip force modulation through the grasping, lifting, holding and release of the instrumented object.

Broadly speaking, excessive grip force has been a notable feature of quantitative research on object manipulation in patients with neurological disorders (52). Hermsdorfer et al. reported particularly important grip force increases for holding, transportation and cyclical vertical movements when using a pinch grip for the hemiparetic arm of stroke patients when compared to the less affected arm. This type of “grip force overshoot” (52) has been interpreted as an increase in the safety margin between the applied force and the minimum force necessary to prevent the object from slipping (49). Large security margins used by stroke patients have previously been associated with the level of somatosensory impairment (37, 48). Nonetheless, Wenzelburger et al. also observed moderate increases in grip force during holding in patients with purely motor capsular stroke (45). In the present study, we observed limited correlation between grip force magnitude with either the FME sensory or motor subscores obtained on the hemiparetic upper limb. Furthermore, consistent with the observation of Nowak et al. (8), a number of patients in the present study also presented with excessive grip forces in their less affected arm (e.g., Figure 8A, patients 1,10,11,12;). Perhaps most striking though was the important variability between patients, with grip force during holding in the range of 4 N−40 N. These vast differences in grip forces underscore the fact that stroke patients are a heterogeneous population and that a clinical presentation of hemiparesis alone is not sufficient for one to presume the magnitude, nor the laterality of changes in grip force scaling. Increased grip force magnitude may reflect compensatory mechanisms in order to compensate for deficits with sensory feedback mechanisms (37, 48) or motor deficits involving poor rate of force development (49). Generalized weakness however may be difficult to discern during lifting and holding as grip forces may be comparable to the grip-load force safety margin.

Issues with the timing of grip force modulation were most notable during the unloading and lifting phases of the task sequence. The increased duration of the grasp time prior to the object being raised from a flat surface is consistent with results from prior studies (10, 13, 49, 66) and reflects the diminished rate of change in grip force during this phase (45, 46, 67). The temporal discrepancy between peak acceleration and maximum grip force observed for the hemiparetic arm in this study is typical of a breakdown in the nervous system's ability to regulate the coupling of grip forces with load forces. McDonnell et al. (46) previously documented a disruption to the coupling between grip and load forces in stroke patients during lifting with a precision grip. The present study expands upon these results, demonstrating that this effect is consistent across the pinch, palmar-digital and top grasps. At the same time, it should be noted that experiments by Hermsdorfer et al. did not observe similar temporal delays when examining cyclical vertical movements (48, 49). This suggests that deficits with temporal coupling for the hemiparetic arm depends upon the type of activity and supports the postulate that motor control for rhythmic motion is relatively distinct from discrete movements (68). Mechanisms for predictive control may be sufficient to regulate grip force load force coupling in regular, continuous alternating movement (48) whereas discrete actions such as lifting would require highly efficient integration of sensory feedback and corresponding muscular adjustments (69). Another (non-exclusive) interpretation is that the lifting and holding task performed by stroke patients with severe impairment is composed of multiple segmented actions and/or may be corrupted by irregularities in proximal control of the arm such that that maximum grip force and acceleration do not coincide.

### Orientation and Stability of the Hand-Held Object

The current body of literature on hand-object orientation in manual dexterity tasks is limited. In the previous study using the iBox with healthy young adults performing the same tasks, Martin-Brevet et al. reported that the object was close to vertical (angle <0.5°) at the times of lifting and placement and marginally more variable during holding (<3°). The values obtained in the present study are considerably higher, particularly during holding. Moreover, significant differences between the hemiparetic and less affected sides were observed (per Figure 6). Whilst not directly measuring object orientation, García Álvarez et al. (53) previously rated object stability for stroke patients when grasping daily objects. They found that object stability was correlated with upper-limb strength (Medical Research Council Scale) and spasticity (Modified Ashworth Scale). Here, quantitative data on iBox orientation resulted in multiple correlations with the Frenchay Arm Test (FAT), although the limited range of scores means caution should be taken with interpretation. Nonetheless, these combined observations suggest that global upper-limb strength is a key factor in regulating the vertical object orientation during lifting, holding and placement tasks.

### Timing and Coordination Errors at Grasp Initiation and Release

The specific design of the instrumented object used in this study allowed us to identify micro errors upon grasp initiation and object release. The rate of these touch and push errors was greater for both the hemiparetic and the less affected side than the rates observed in healthy young adults (57). The increased frequency of push errors during lifting here is generally consistent with the observations of McDonnel et al. (46). Similarly, Duque et al. (44) observed a greater duration between the first touch by the thumb or index and the onset of grasp forces for children with cerebral palsy when compared to age-matched controls. These kinds of touch errors may be seen as evidence of an impairment in the transition between reach and grasp. We would suggest that the apparent lack of synchrony between thumb and finger movement as they close upon or withdraw from an object may be associated with the hand and palmar arch pre-shaping deficits previously documented by Sangole et al. (70).

### Effect of Grasp Configuration

The effects of hand configuration upon grasp regulation during lifting, holding and object placement represents the central finding of the present study. As hypothesized, the use of the different grasps (precision, top, pinch, palmar-digital) had important effects upon the magnitude and timing of grip force adjustments, object orientation as well as the frequency of errors. Most notably, grip forces were greatest when using the palmar-digital grasp. This observation is consistent with prior results in healthy adult subjects (57). Whilst coupling between grip forces and load forces was apparent across all the grasp combinations, the time delay between maximum grip and peak acceleration was greater in the palmar-digital grasp than the top grasp. In an experimental paradigm involving cyclic vertical movements, Flanagan and Tresilian similarly observed temporal delays in the coordination between grip forces and load forces when using a palmar-digital grasp (34). They suggested that these differences may reflect diminished tactile information in certain parts of the hand. A lower density of glabrous skin receptors through the palm than in the thumb and fingers may limit the precision of fine tuning abilities (32). The increased grip force observed in palmar-digital grasp would thus represent an increased safety margin to account for this limitation. In the present study, we found that the frequency of touch errors was greatest when initiating a palmar-digital grasp and that this grasp configuration was associated with variable object orientation at *tl*. Importantly, stroke patients with more important impairments tend to use palmar-digital grasp configurations more consistently than less impaired stroke patients or healthy adults (53). Therefore, whilst this behavior may assist stroke patients to compensate for reduced dexterity or muscle strength (53), the results presented here indicate that this preferential use of the palmar-digital grasping strategies may impact upon task execution in terms of grip force economy, temporal precision of grip force adjustments, and stability of the hand-held object.

In contrast to this, the top grasp configuration was associated with lower grip forces and comparably lower temporal discrepancies between peak acceleration and maximal grip force. The increased levels of wrist flexion when using the top grasp configuration may contribute to these differences. In healthy subjects, maximum grip-strength varies according to wrist position (71–73) and influences grip force regulation (74). Of course, when in an extended position, extrinsic flexors of the wrist and fingers are stretched, and conversely, a flexed position brings about passive finger extension (tenodesis effect). Increased flexor tone is common following a stroke, hence this effect may be exaggerated (75). Additionally, it has been proposed that the modification of afferent input associated with the changes in muscle length across the wrist could affect cortical and spinal excitability (74). Allowing a stroke patient to use a top grasp may thus limit these passive increases in muscle tension and further inhibit (excessive) neurological drive. Regardless of the precise mechanisms involved, the increased temporal precision of grip force adjustments when using a top grasp may be informative in clinical practice. It would suggest that use of top grasp hand configurations may be an adaptive strategy to assist stroke patients with tasks specifically requiring responsive grip force adjustments.

### Effects of Side

Differences in grasp regulation between the hemiparetic and less affected arms were observed most notably in the frequency of errors at grasp onset, the duration of the unloading phase and object angle at lifting and placement. Interestingly, the frequency of touch errors on grasping with the hemiparetic side was associated with sub-threshold finger contact, whereas in the less affected arm, touches they were more frequently associated with sub-threshold thumb contact. This appears consistent with previously described kinematic patterns where patients move their hand around an object in the approach phase, a strategy which may serve to compensate for weakness in the wrist extensors and/or finger flexors (54, 76). In other terms, this could be thought of as “leading with the fingers” in preparing for object handling with the hemiparetic arm as opposed to “leading with the thumb” when preparing for object handling with the less affected arm. Release phase transitions were also characterized by asynchrony between the thumb and fingers on the hemiparetic side. Certain studies have suggested that this type of issue is linked to a distinct impairment of the grasp release mechanisms (77, 78). At the same time, such an error could also conceivably be hindered by limitations with proximal control as the patient attempts to withdraw their hand. Future studies should seek to combine kinematic analysis of upper-limb movement with measures from instrumented objects in order to understand patterns of coordination across the arm, hand and object as an ensemble. Finally, as evoked above (section Orientation and Stability of the Hand-Held Object), it is likely that upper-limb strength is important for maintaining vertical object orientation. The specific increases in the variability of object orientation at *tl* and *tp* seen in the hemiparetic arm (per Figure 6) further suggest that patients have the greatest difficulty maintaining object stability in the transition of the object to and from the working surface.

## Limitations of the Study

The principal limitation in the design of this study is the lack of control group. Whilst one of our previous studies involved a similar protocol, data was obtained only for young adults. In the absence of an age matched control group we have limited our analysis to differences in grasp regulation between the hemiparetic and less affected arms of patients following a stroke. Secondly, whilst the iBox affords certain advantages (ease of manipulation, multiple integrated sensors), it measures exlusively those forces normal to the surface of each face—it is unable to estimate tangential forces or torque. The choice for linear load cells was motivated by the possibility of an affordable object which could be used in the clinic or at home (58).

Finally, the design of this study allows for considerable variation in surface contact. The coefficient of friction between a hand and an object varies according to the properties of a subject's skin (79) and the texture of the object (31). Increasing surface contact increases the coefficient of friction (80), a factor which was not controlled for in this experiment from one grasp configuration to the next. Consequently, the analysis of force exchanges with the iBox has certain limits for comparison across the grasping strategies. It is interesting to note however, that the subjects employed greater grasp forces when using the palmar-digital grasp despite having a greater coefficient of friction. This underscores that grip force regulation is contingent upon numerous biomechanical and neurological variables. In the present study, we consider the measurable behaviors as representative of the strategies associated with each grasp configuration.

## Conclusion and Perspectives

To surmise, the magnitude and temporal precision of grip force adjustments varied according to the different grasp configurations employed by hemiparetic patients. More specifically, grip forces were consistently greatest when patients used a palmar-digital grasp and lowest when using a top grasp. Similarly, the time delay between peak acceleration and maximum grip during lifting were highest in palmar-digital grasp and lowest top grasp. Use of the hemiparetic arm resulted in greater variability in the vertical orientation of the object, in particular upon lifting the object from and placing the object upon the working surface. Both grasp configuration and use of the hemiparetic arm were found to contribute to the occurrence of touch and push errors when establishing grasp or releasing the object. Our interpretation of this is that structural aspects of hand configuration contribute considerably to the grip force scaling while the effects of hemiparesis on upper-limb coordination more globally bring about deficits with object control and orientation at transitions in task sequence such as grasp onset, lifting, object placement and release.

These observations may assist in understanding the functional implications of compensatory grasp strategies in patients with hemiparesis and assist with facilitating adaptive prehension patterns in the context of rehabilitation. That is to say, whilst patients suffering from stroke may have exhibit preferences for taking objects with palmar-digital grasp configurations (53), this strategy may have negative effects upon grip force economy and temporal precision of grip force adjustments. The use of top grasp may thus be indicated in order to facilitate more responsive control in day to day object handling for this population.

## Author Contributions

RP, SM, and PP-D were responsible for patient recruitment. Data collection and patient evaluations were carried out by RP and SM. The iBox device and software was designed and adapted by NJ. Data analysis was performed by RP, NJ, and AR-B while VM-P participated in the discussions. The manuscript was drafted by RP and AR-B. All authors participated in the revision of the manuscript.

### Conflict of Interest Statement

The authors declare that the research was conducted in the absence of any commercial or financial relationships that could be construed as a potential conflict of interest.

## Footnotes

^1^Probably due to the design of the iBox, we could not distinguish a first phase of increasing grasping force without change in vertical force (see inset of Figure 3). The unloading period in this work corresponds to the sum of pre-loading and loading periods commonly identified in previous studies where the vertical force sensor is fitted between the handle of the manipulandum and its main mass.
