# Supplementary material for: Effects of Hand Configuration on the Grasping, Holding, and Placement of an Instrumented Object in Patients With Hemiparesis
Source: Front Neurol. 2019 Mar 19;10:240. doi: 10.3389/fneur.2019.00240 (PMC6433942; doi:10.3389/fneur.2019.00240)
Supplement: Supplementary file 1 [file Data_Sheet_1.pdf]

frontiers

Supplementary material to Parry et al.

Effects of Hand Configuration on the Grasping, Holding and Placement of an Instrumented Object in Patients With Hemiparesis

Tables S1—S6 provide a summary of significant correlations between clinical evaluations and iBox parameters measured across the four different hand configurations used. Values in bold represent the spearman correlation coefficients while the values in parentheses indicate the 95% confidence intervals for the correlation matrices detailed in section 2.8 of the manuscript. Columns of each table have been shaded in order to distinguish temporal parameters (green), grip force parameters (red), object angle (blue) and contact errors (grey).

Table S1: Correlation between strength measured with grip force dynamometer (DGS) and variables measured with iBox

	Force change during unloading	Force change during release	Max grip force	Hold force average	Grip force at *tp*	Alpha at *tl*
**Precision**	ns	ns	ns	**0.738** [-0.714/0.714]	**0.738** [-0.714/0.714]	ns
**Top**	ns	ns	ns	ns	ns	**-0.718** [-.618/0.600]
**Pinch**	ns	ns	ns	ns	ns	ns
**Palmar**	**0.720** [-0.580/0.594]	ns	**0.762** [-0.580/0.577]	**0.867** [-0.573/0.580]	ns	ns

Table S2: Correlation between Fugl Meyer sensory function subscore and variables measures with iBox

	Release phase duration	Force change during unloading	Force change during release	Grip force at *tl*	Max grip force	Hold force average	Grip force at placement	Grip force at *tr*	Alpha at *tl*	Alpha during hold phase
**Precision**	**-0.743** [0.719/0.719]	**0.743** [-0.719/0.730]	**0.755** [-0.719/0.731]	**0.779** [-0.707/0.719]	**0.779** [-0.719/0.707]	**0.802** [-0.719/0.731	**0.802** [-0.719/0.731]	ns	ns	ns
**Top**	ns	ns	ns	ns	ns	ns	ns	ns	ns	ns
**Pinch**	**-0.7306** [-0.616/0.594]	ns	**0.781** [-0612/0.603]	ns	ns	ns	ns	**-0.731** [-0.608/0.603]	ns	ns
**Palmar**	ns	ns	ns	ns	ns	ns	ns	ns	**-0.799** [-0.646/0.657]	**-0.835** [-0.630/0.6383]

Table S3: Correlation between Fugl Meyer motor function subscore and variables measures with iBox

	Duration of unloading phase	Release phase duration	Force change during unloading	Force change during release	Peak acceleration	Grip force at *tr*	Alpha during hold phase
**Precision**	**-0.731** [-0.731/0.719]	**-0.779** [-0.719/0.707]	ns	ns	**0.826** [-0.719/0.7186]	ns	**-0.728** [-0.634/646]
**Top**	**-0.736** [-0.565/0.576]	**-0.766** [-0.580/0.577]	ns	ns	ns	ns	ns
**Pinch**	ns	**-.705** [-0.613/0.604]	ns	ns	ns	**-0.728** [-0.604/0.613]	
**Palmar**	**-0.805** [-0.585/0.573]	**-0.805** [-0.584/0.587]	**0.8787** [-0.587/587]	**0.714** [-0.569/0.587]	ns	ns	ns

Table S4: Correlation between Fugl Meyer Evaluation (FME) total score and variables measured with iBox

	Unloading phase duration	Release phase duration	Force change during unloading	Force change during release	Maximal grip force	Grip force at *tr*	Alpha at *tl*	Alpha during hold phase	Alpha at *tp*
Precision	**-0.862** [-0.731/0.731]	**-0.838** [-0.719/0.719]	**0.814** [-0.707/0.719]	**0.755** [-0.731/0.719]	ns	ns	ns	**-0.829** [-0.628/0.638]	**-0.757** [-0.757/0.775]
Top	ns	ns	ns	ns	ns	ns	ns	ns	ns
Pinch	ns	**-0.790** [-0.612/0.598]	ns	ns	ns	**-0.872** [-0.598/0.607]	ns	ns	ns
Palmar	**-0.842** [-0.579/0.591]	**-0.758** [-0.596/0.589]	**0.867** [-0.579/0.582]	**0.709** [-0.586/0.590]	**0.702** [-0.584/586]	ns	**-0.811** [-0.639/0.644]	ns	ns

Table S5: Correlation between Jebsen Taylor Hand Function Test (JTT) and variables measures with iBox

	Duration of unloading phase	Release phase duration	Force change during unloading	Force change during release	Lag time (max grip to peak acc)	Grip force at *tl*	Alpha at *tg*	Alpha during hold phase	Alpha at *tp*
**Precision**	ns	ns	ns	ns	ns	ns	ns	ns	ns
**Top**	**0.833** [-0.714/0.716]		**-0.762** [-0.714/0.714]	ns	ns	**0.810** [-0.714/0.714]	**0.762** [-0.75/0.75]	ns	ns
**Pinch**	ns	**0.786** [-0.714/0.738]	**-0.714** [-0.714/0.714]	**-0.810** [-0.714/0.714]	**0.738** [-0.714/0.714]	ns	ns	ns	**0.786** [-0.714/0.714]
**Palmar**	ns	ns	ns	ns	ns	ns	ns	**0.976** [-0.771/0.771]	ns

Table S6: Correlation between Frenchay Arm Test (FAT) and variables measured with iBox

	On touches	Force change during unloading	Grip force at *tg*	Alpha at *tl*	Alpha during hold phase	Alpha variance during hold
**Precision**	ns	ns	ns	ns	ns	ns
**Top**	**-0.724** [-0.584/0.589]	**0.706** [-0.582/0.579]	**-0.740** [-0.572/0.582]	**-0.703** [-0.598/0.621]	ns	**-0.846** [-0.632/0.640]
**Pinch**	ns	ns	ns	**-0.780** [-0.611/0.616]	ns	ns
**Palmar**	ns	ns	ns	ns	**-0.787** [-0.628/0.638]	**-0.787** [-0.637/0.634]
